# WBP2 negatively regulates the Hippo pathway by competitively binding to WWC3 with LATS1 to promote non-small cell lung cancer progression

**DOI:** 10.1038/s41419-021-03600-3

**Published:** 2021-04-09

**Authors:** Qiang Han, Xuezhu Rong, Xuyong Lin, Xiupeng Zhang, Chuifeng Fan, Huanyu Zhao, Enhua Wang

**Affiliations:** 1 Department of Pathology, College of Basic Medical Sciences and the First Affiliated Hospital of China Medical University, Shenyang, China; 2 Department of Pathology, the First Affiliated Hospital of China Medical University, Shenyang, China

**Keywords:** Cancer, Tumour biomarkers

## Abstract

WW domain binding protein-2 (WBP2) can function as a Yes-associated protein/transcriptional co-activator with PDZ-binding motif (YAP/TAZ) co-activator and has a crucial role in promoting breast cancer progression. However, the expression and potential molecular mechanisms of WBP2 in the context of lung cancer are not fully understood. We determined that WBP2 was highly expressed in lung cancer specimens and cell lines and that this expression was closely related to the advanced pTNM stage, lymph node metastasis, and poor prognosis of patients. In addition, gain- and loss-of-function experiments revealed that WBP2 could significantly promote the proliferation and invasion of lung cancer cells both in vivo and in vitro. To elucidate the underlying molecular mechanism, we determined that wild-type WBP2 could competitively bind to the WW domain of WWC3 (WW and C2 domain-containing-3) with LATS1 (Large tumor suppressor-1) through its PPxY motifs, thus inhibiting the formation of the WWC3-LATS1 complex, reducing the phosphorylation level of LATS1, suppressing the activity of the Hippo pathway, and ultimately promoting YAP nuclear translocation. Therefore, from the aspect of upstream molecules of Hippo signaling, WBP2 promotes the malignant phenotype of lung cancer cells in a unique manner that is not directly dependent upon YAP, thus providing a corresponding experimental basis for the development of targeted therapeutic drugs for lung cancer.

## Introduction

The Hippo pathway, initially identified in Drosophila, is highly conserved across the evolution of species, playing a crucial role in maintaining homeostasis, regulating cell proliferation, differentiation, and other physiological processes. Studies have confirmed that disorders associated with Hippo activity lead to tumor progression^1,2^. On activation of the classical Hippo pathway, the upstream molecules induce the phosphorylation of the central kinase MST-LATS (mammalian sterile 20-like kinase/large tumor suppressor) complex, promoting the phosphorylation of YAP. This protein remains in the cytoplasm, binds to the 14-3-3 protein, and is then degraded by the ubiquitin-proteasome system. However, when the Hippo pathway is inhibited, YAP escapes the protease and accumulates within the cytoplasm, translocating into the nucleus to bind to the transcription factor TEADs to activate transcription of Hippo target genes (*CTGF* and *Cyr61*)^3–5^.

WW domain binding protein-2 (WBP2) was initially identified as a homologous ligand-protein of the YAP–WW domain and found to interact with the paired box gene-8 (PAX8) transcription factor with unknown function^6–9^. Several studies have focused on the molecular function of WBP2 in human solid tumors by assessing the impact of this protein on the biological phenotype^10–15^. However, both the expression pattern of WBP2 in lung cancer and the ability of this protein to regulate the activity of the Hippo pathway in an indirect manner that is dependent upon YAP remain unreported.

WW and C2 domain-containing protein-3 (WWC3) belongs to the WWC family (KIBRA/WWC1, WWC2, and WWC3), which serves as the classic upstream protein molecule in the Hippo pathway. Kremerskothen and colleagues^16,17^ from Muenster University have observed that WWCs could activate Hippo pathway activity by binding to the PPxY motif of LATS kinase via the WW domain, promoting LATS auto-phosphorylation. Our previous studies have further demonstrated that WWC3 regulates the activities of both the Hippo and Wnt pathways primarily through interactions with LATS and DVLs, which is facilitated by the WW domain, and that WWC3 plays an important role in inhibiting the malignant phenotype of lung cancer^18,19^.

In this study, we attempted to verify WBP2 expression in clinical samples and cell lines, and then explored the underlying molecular mechanism involved in non-small cell lung cancer (NSCLC), providing an experimental basis for the identification of molecular markers of lung cancer and the development of targeted therapeutic agents.

## Material and methods

### Patient information and specimens

This study was approved by the ethics committee of the China Medical University and was performed according to the requirements of the Declaration of Helsinki. All patients with lung cancer who participated in this study were aware of the study and signed an informed consent form. A total of 127 lung cancer specimens and 32 normal lung tissue samples were collected from the Department of Pathology of the First Affiliated Clinical Hospital of China Medical University. None of the patients received chemotherapy or radiotherapy before the operation. According to the 2015 lung cancer classification standard^20^, 99 cases were classified as stage I–II and 28 cases were classified as stage III, 73 and 54 patients presented with adenocarcinoma and squamous cell carcinoma, respectively. Complete follow-up data were obtained for 93 patients. According to the percentage of cells stained, the expression of WBP2 was divided into five grades: 0 (no staining), 1 (1–25%), 2 (26–50%), 3 (51–75%), and 4 (>75%). Two investigators, who were blinded to the clinical data, examined all tumor slides. Five random fields of view were examined per slide. According to the intensity of cell staining, the expression of WBP2 was divided into three grades: 0 (no staining), 1 (light yellow particles), 2 (moderate staining), and 3 (yellow–brown particles). The scores were multiplied to give a final score of 0–12. The expression intensity of WBP2 in most cases was >4. Therefore, we defined the expression of WBP2 that was <4 as negative expression (−), whereas 4–5 was considered weakly positive (+), 6–7 was moderately positive (++), and ≥7 was strongly positive (+++).

### Immunohistochemistry

Assays were performed as described previously^19^. Slides were incubated overnight with polyclonal rabbit-derived WBP2 antibody (HPA065682, 1:50, Sigma-Aldrich, St. Louis, MO, USA) at 4°C. Detailed information is provided in the Supplementary Material and Methods.

### Western blot, co-immunoprecipitation

Assays were performed as described previously^19^. The primary antibodies used for these experiments are listed in the Supplementary Material and Methods. Immunoreactive bands were detected with electrochemiluminescence; the expression of GAPDH was used as the relative loading control.

### GST-pulldown

The WWC3 protein coupled to a GST label was induced in *Escherichia coli* BL21 (30° C, 3 h, 200 rpm/min) and purified according to standard steps. The purified protein was recombined with glutathione sepharose (GE Healthcare, Waukesha, WI, USA) magnetic beads and then incubated overnight with H1299 cell lysate transfected with Myc-WBP2 plasmid at 4°C. Finally, the complexes were detected by western blotting and Coomassie brilliant blue staining.

### Cell lines

The immortalized human bronchial epithelial cell line (HBE) was purchased from the American Type Culture Collection (ATCC, Manassas, VA, USA). The human NSCLC lines H661, H1299, A549, and Calu-1 were purchased from Shanghai Cell Bank of the Chinese Academy of Sciences (Shanghai, China). The LK2 cell line was kindly provided by Prof. Hiroshi Kijima (Department of Pathology and Bioscience, Hirosaki University Graduate School of Medicine, Japan). All cells were cultured in either Dulbecco’s modified Eagle medium (DMEM, Hyclone, Logan, UT, USA) or Roswell Park Memorial Institute-1640 (RPMI-1640, Hyclone) medium supplemented with 10% fetal bovine serum (Hyclone) at 37°C in a humidified atmosphere at 5% CO_2_. All cell lines were authenticated using short tandem repeat DNA profiling and tested for mycoplasma contamination.

### Plasmid construction and transfection

Lipofectamine 3000 (Invitrogen, Carlsbad, CA, USA) was used for transient transfection. Stable transfection was screened for using purinomycin and G418 (Sigma-Aldrich). The plasmids and small interfering RNA used are listed in the Supplementary Material and Methods.

### Immunofluorescence assay

Assays were performed as described previously^19^. The primary antibodies included WBP2 antibody (#HPA065682, 1:25, Sigma-Aldrich), WWC3 antibody (#HPA039814, 1:50, Sigma-Aldrich), and YAP antibody (#14074, 1:50, Cell Signaling Technology, Danvers, MA, USA).

### Colony formation experiment, cell migration, and matrix invasion assays

The cells were transfected for 48 h and then inoculated into a 6 cm cell culture dish (1000 cells/plate) and incubated for 12 days. The cells were then washed with phosphate-buffered saline (three times for 5 min) and subsequently stained with hematoxylin for 10 min before counts were performed; the experiments were repeated in triplicate to acquire an average value.

For the matrix invasion assay, the matrix adhesive (BD Biosciences, CA, USA) was diluted in a 24-well plate at a 1:3 ratio in DMEM that was free of fetal serum and antibiotics. The lung cancer cells were inoculated in the upper chamber at a density of 5 × 10^5^ cells in 100 μL of medium without fetal serum, and culture medium containing 10% fetal serum (Hyclone) was placed in the lower chamber. After 24 h of cultivation, cells were fixed for 15 min using methanol, and hematoxylin staining was subsequently performed. Ten fields were randomly selected to count the number of invading cells. The experiment was repeated in triplicate, and the average value was acquired accordingly.

### RNA extraction and quantitative PCR

Assays were performed as described previously^19^. The experiments were performed in triplicate. The primer sequences are listed in Supplementary Material and Methods.

### Dual-luciferase reporter genes assay

The assays were performed according to the manufacturer’s protocol (Promega, Madison, Wisconsin, USA). YAP/TEAD transcriptional activity was measured using a luciferase assay based on the pGL3b_8xGTIIC-luciferase plasmid, which contains the TEAD consensus in the vector, obtained from Addgene (plasmid #34615, Cambridge, MA, USA). Cells were transfected to express the indicated proteins and *Renilla* luciferase was used as a control for signal normalization. Six independent transfections were performed for each experiment. The data were normalized to those of the empty vector control and are presented as the average ± SD.

### Nuclear and cytosolic fractionation

Assays were performed as described previously^19^. Beta-tubulin (sc-166729, 1:500, Santa Cruz Technology) and LaminB1 (sc-374051, 1:500, Santa Cruz Technology) were used as the cytosolic and nuclear loading control, respectively.

### Animal experiments

For the subcutaneous tumor formation experiment, a total of 12 BALB/c nude mice (three BALB/c nude mice per group, 4 weeks old, female, 16–20 g, specific pathogen-free standard) were purchased from Beijing Charles River Company (Beijing, China). Food and drinking water were sterilized using a semi-barrier system at constant temperature and humidity. Each nude mouse was randomly assigned to each group and the investigator was blinded to the group allocation. All animal experiments were performed in accordance with the ethical regulations of animal experiments at the China Medical University. The cell concentration for each group was adjusted to 5 × 10^6^ cells/mL, and 0.2 mL of cells was injected subcutaneously into the backs of nude mice. After 4 weeks of observation beginning from the day of injection, the mice were killed, and the weights and volumes of the subcutaneous tumors were recorded accordingly.

For the intrapulmonary metastasis experiment (28 BALB/c nude mice, 7 BALB/c nude mice per group), the lungs were removed and fixed in 10% neutral formalin. Paraffin-embedded sections were prepared and stained with hematoxylin and eosin. The numbers and sizes of the metastatic foci in the lungs were recorded.

### Statistical analysis

All experiments were repeated at least in triplicate. All data were analyzed using SPSS 22.0 (SPSS, Inc., Chicago, IL, USA). Chi-square tests were used to test the correlation between WBP2 expression and clinicopathological factors. All clinicopathological parameters were included in the Cox regression model and tested by univariate analysis using the enter method and multivariate analysis using the forward stepwise logistic regression method. The Student’s *t* test was used to analyze differences between groups. A *P* value <0.05 (two-sided) was considered statistically significant.

## Results

### WBP2 is highly expressed in NSCLC and is associated with poor prognosis

**Fig. 1:**
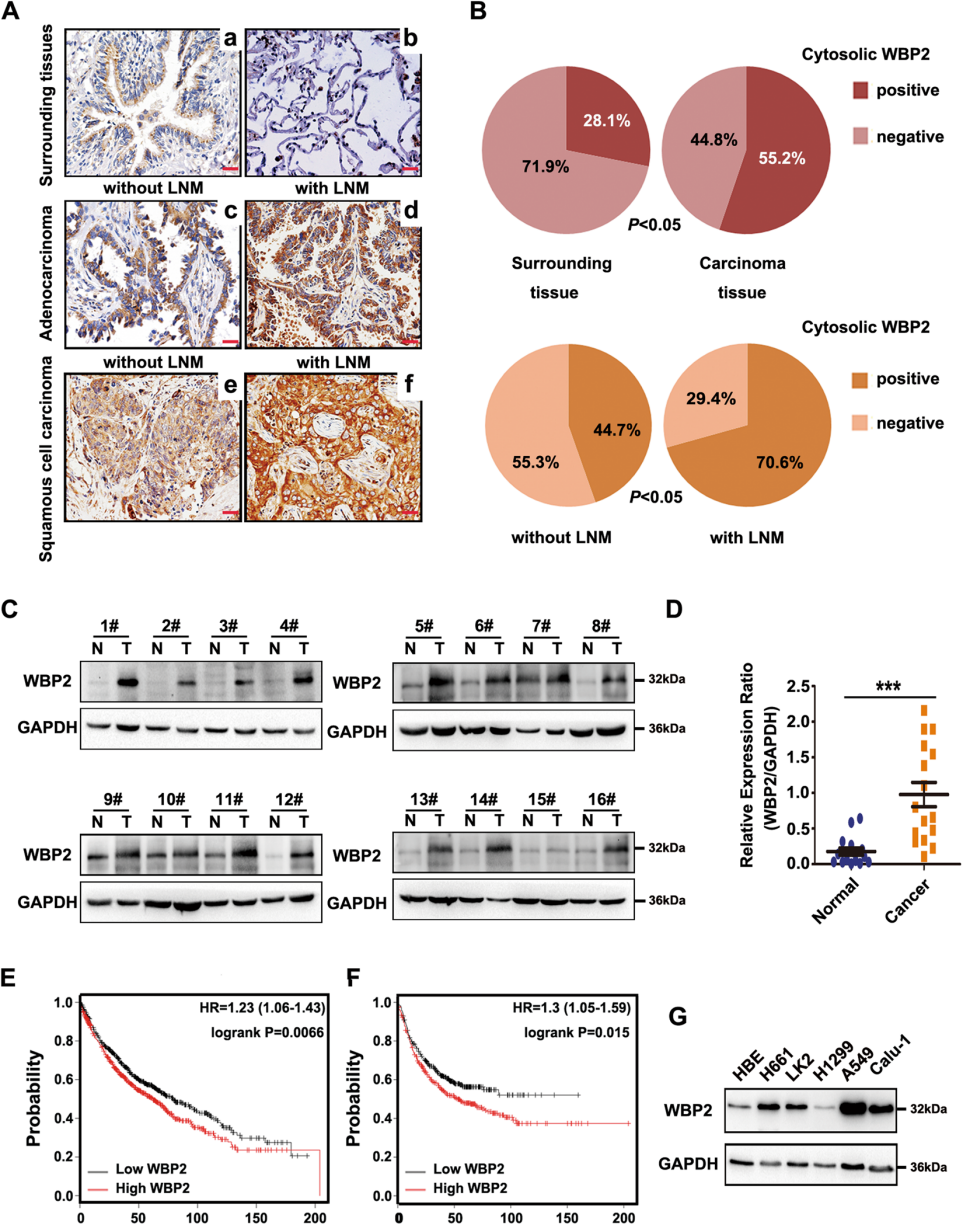
High expression levels of WBP2 in non-small cell lung cancer correlate with poor prognosis. **A**, **B** The expression of WBP2 is low or negative in normal bronchial epithelium (**A**–**a**) and alveolar epithelium (**A**–**b**), positive (+−++) in adenocarcinoma (**A**–**c**) and squamous cell carcinoma (**A**–**e**) without lymph node metastasis (LNM), and strongly positive (++−+++) in adenocarcinoma (**A**–**d**) and squamous cell carcinoma (**A**–**f**) with LNM. Magnification: ×400, scale bar: 50 μm. LNM: lymph node metastasis. **C**, **D** Western blot analysis revealed that the expression level of WBP2 in lung cancer tissues is significantly higher than that in adjacent normal lung tissues. GAPDH was used as the loading control. **E**, **F** Kaplan–Meier plotter network database analysis indicates that the overall survival (OS; **E**) and progression-free survival (PFS; **F**) of patients with lung cancer presenting high WBP2 expression are significantly lower than those of patients with low WBP2 expression. **G** Western blot analysis of WBP2 expression in the human immortalized bronchial epithelial cell line HBE and in a panel of non-small cell lung cancer cell lines (*n* = 5). GAPDH was used as the loading control. *P* < 0.05 indicates statistical significance, and *** represents *P* < 0.001.

To determine whether WBP2 has a specific role in NSCLC, we first detected the expression of WBP2 in lung cancer specimens, and further assessed the association of this expression with the survival and prognosis of patients via immunohistochemistry and immunofluorescence staining. The results indicated that WBP2 was localized in the cytoplasm of lung cancer cells, whereas with regard to expression, WBP2 was poorly or even negatively expressed in normal cells (71.9%, 23/32) (low expression in normal bronchial epithelial cells, negative expression in normal alveolar epithelium), but highly expressed in lung adenocarcinoma and squamous cell carcinoma (55.2%, 70/127, Fig. 1A, Supplementary Fig. S1). In addition, the difference in expression between para-cancerous and cancerous tissues was significant (*P* < 0.05, Fig. 1B). Notably, WBP2 expression in patients with lymph node metastasis was significantly higher than that observed in patients without lymph node metastasis (44.7 vs 70.6%, *P* < 0.05, Fig. 1A, B). The Chi-square test revealed that high WBP2 expression was closely related to the advanced pTNM stage (*P* = 0.001) and positive lymph node metastasis (*P* = 0.006) in patients with NSCLC (Table 1). Cox univariate and multivariate analyses indicated that a high TNM stage, adenocarcinoma histological type, and high WBP2 expression (*P* = 0.038, *P* = 0.017, and *P* = 0.030, respectively; Table 2) were all independent prognostic factors in NSCLC. Accordingly, western blot analyses revealed that the expression level of WBP2 was significantly higher in lung cancer tissues than that observed in adjacent tissues (14/16, Fig. 1C, D, *P* < 0.05). The online network database (http://www.kmplot.com) suggested that high WBP2 expression was negatively correlated with overall survival and progression-free survival (*P* = 0.0066 and *P* = 0.015, respectively; Fig. 1E, F). Furthermore, the Kaplan–Meier survival analysis verified these results (*P* < 0.05, Supplementary Fig. S2). Compared with the normal bronchial epithelial cell line HBE, WBP2 was highly expressed in the four lung cancer cell lines (Fig. 1E, *n* = 5). This finding was consistent with results obtained using clinical tissue specimens. Accordingly, it can be suggested that WBP2 may play a role in promoting malignancy by functioning as an oncogene.

**Table 1:** Association of WW domain binding protein-2 (WBP2) expression with clinical and pathological characteristics in non-small cell lung cancer (NSCLC).

Clinicopathological characteristics	*N*	Positive	Negative	*χ*^2^	*P* (two-sided)
Age (years)					
<60	37	25	12	3.271	0.080
≥60	90	45	45		
*Gender*					
Male	79	43	36	0.040	0.856
Female	48	27	21		
Histological type					
Squamous cell carcinoma	54	32	22	0.651	0.473
Adenocarcinoma	73	38	35		
Differentiation					
Well	40	22	18	0.000	1.000
Moderate & poor	87	48	39		
TNM classification					
I + II	99	47	52	10.604	0.001*
III	28	23	5		
Lymph node metastasis					
Positive	51	36	15	8.245	0.006*
Negative	76	34	42		

*P < 0.05, statistically significant.

**Table 2:** Summary of Cox univariate and multivariate regression analysis of the association between clinicopathological characteristics and overall survival in 93 cases of non-small cell lung cancer.

Clinicopathological characteristics	Hazard ratio (95% CI)	*P*
Univariate analysis		
Age older than 60 years	0.534 (0.286–1.032)	0.062
Gender: male	0.973 (0.515–1.839)	0.934
Histological type: adenocarcinoma	0.450 (0.237–0.855)	0.015*
Poor differentiation	1.906 (0.968-–3.754)	0.062
High TNM classification	3.768 (1.908–7.444)	0.000*
Positive lymph node metastasis	2.713 (1.445–5.096)	0.002*
Positive WBP2 expression	3.274 (1.599–6.701)	0.001*
Multivariate analysis		
Histological type: adenocarcinoma	0.431 (0.216–0.860)	0.017*
High TNM classification	2.756 (1.058–7.179)	0.038*
Positive WBP2 expression	2.294 (1.082–4.867)	0.030*

*P < 0.05, statistically significant.

### Ectopic expression of WBP2 promotes proliferation, migration, and invasion of NSCLC cells both in vitro and in vivo

**Fig. 2:**
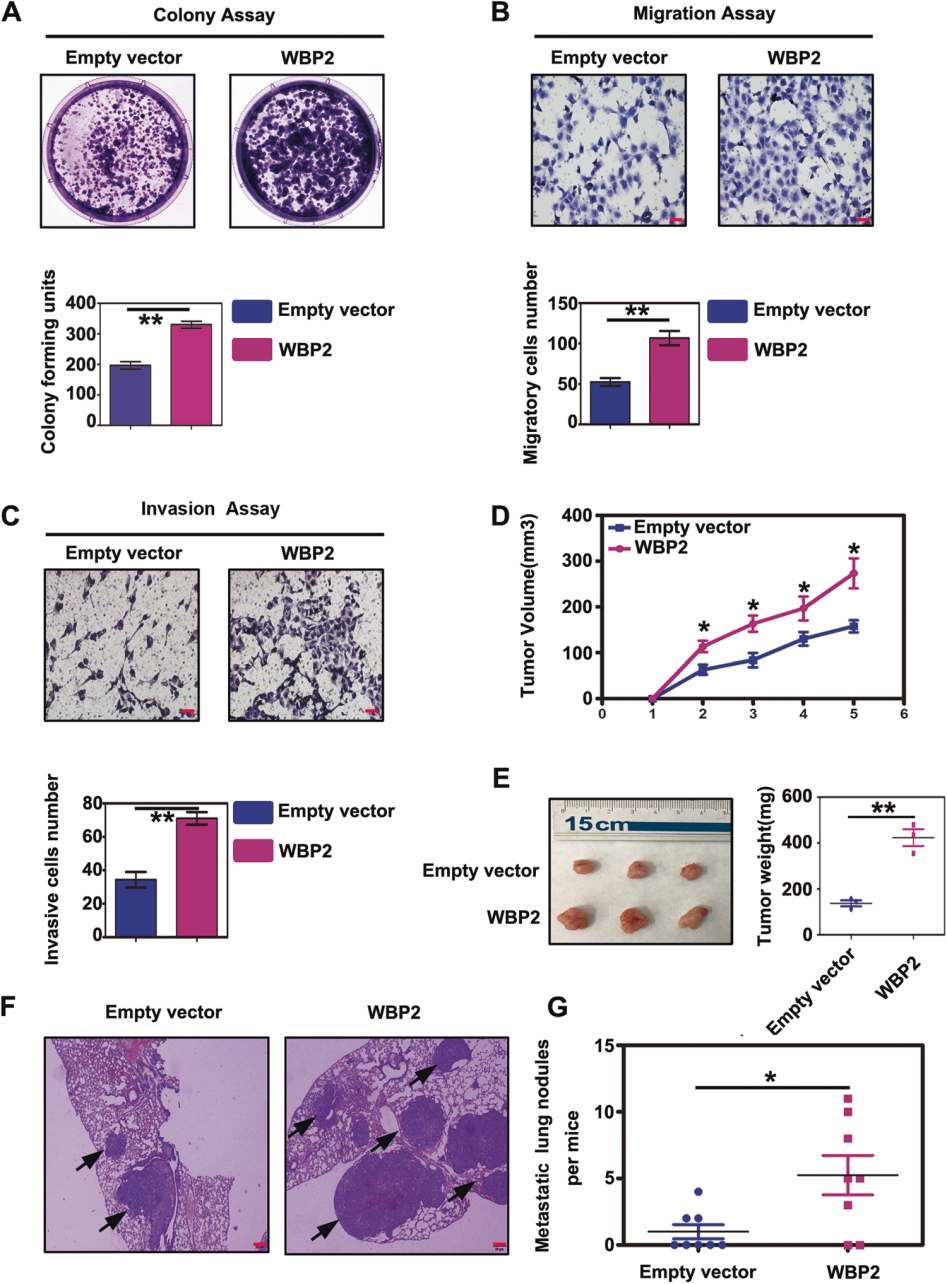
Overexpression of WBP2 promotes proliferation, invasion, and metastasis of lung cancer cells. **A–C**, In vivo experiments: the plasmid WBP2 was transfected into the H1299 cell line, and stable expression monoclonal cells were screened using G418 (800 μg/mL). Colony-forming assays and transwell assays revealed that overexpression of WBP2 protein significantly enhances the proliferation (**A** control vs WBP2: 196 ± 12 vs. 330 ± 12, *P* < 0.01), migration (**B** control vs. WBP2: 52 ± 5 vs. 106 ± 8, *P* < 0.01), and invasiveness (**C** control vs. WBP2: 34 ± 4 vs. 71 ± 3, *P* < 0.01) of lung cancer cells. **D**–**G** Subcutaneous tumor transplantation and lung metastasis experiments via tail vein injections in nude mice demonstrate that the volumes and weights of subcutaneous transplanted tumors in nude mice are both significantly increased following WBP2 overexpression (**D**, **E** Volume:control vs WBP2: 157.7 ± 13.0 vs 273.3 ± 32.8 [mm^3^], *P* < 0.05; weight:control vs WBP2: 137.3 ± 13.1 vs 423.3 ± 36.5 [mg], *P* < 0.01). The number of lung cancer metastasis foci is also markedly increased (**F**, **G** control vs WBP2: 1.0 ± 0.5 vs 5.2 ± 1.5, *P* < 0.05). *P* < 0.05 indicates statistical significance, *P < 0.05, **P < 0.01.

We attempted to determine whether WBP2 impacts the malignant phenotype of tumor cells in NSCLC. As shown in Fig. 1G, WBP2 expression was the highest in A549 cells, but was relatively low in H1299 cells. Therefore, we selected the H1299 cell line for the overexpression experiments. The colony formation assay revealed that, compared with the control group, the proliferative abilities of H1299 cells were significantly enhanced after stable transfection of WBP2 (Fig. 2A). In addition, we observed that WBP2 overexpression promoted the migration and invasiveness of lung cancer cells (Fig. 2B, C). These experimental results indicated that WBP2 possessed the ability to promote malignant phenotypes of tumors in vitro. To further verify whether WBP2 exerts similar effects in vivo, we performed subcutaneous tumor transplantation and lung metastasis experiments following tail vein injections in nude mice. Compared with the control group, the volumes and weights of the subcutaneous tumors (Fig. 2D, E) and the number of metastatic foci (Fig. 2F, G) in the lungs of mice stably overexpressing WBP2 were significantly increased; these findings were consistent with results obtained in vitro.

### WBP2 knockdown weakens the malignant phenotype of lung cancer cells both in vivo and in vitro

**Fig. 3:**
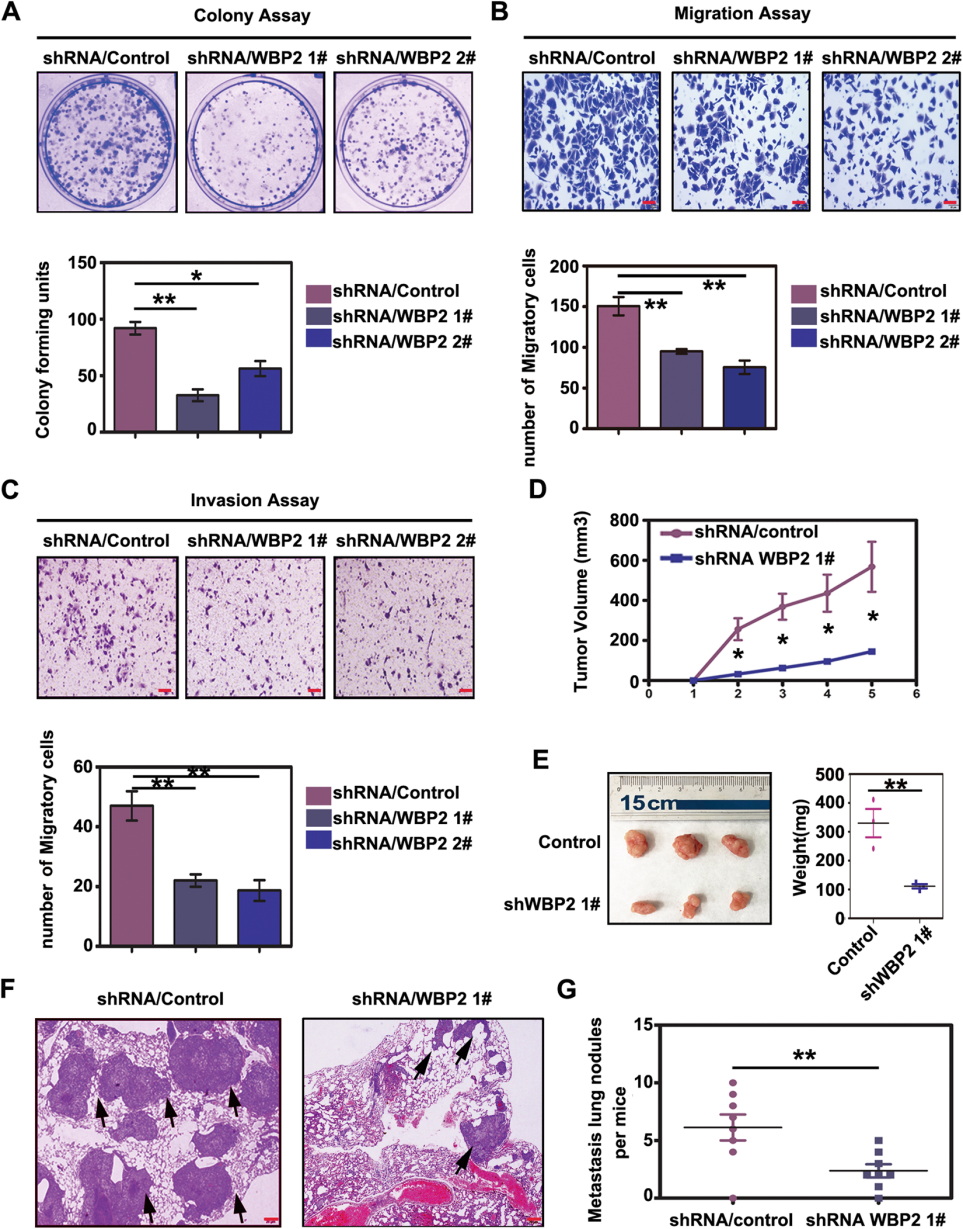
Knockdown of WBP2 weakens the proliferation, invasion, and metastatic abilities of lung cancer cells. Lentivirus-coated shRNA-WBP2 was added to the A549 cell line, and stable cells with WBP2 knockdown were screened for use of puromycin (5 μg/mL). The colony-forming and Transwell assay results reveal that the reduction in WBP2 protein significantly weakens proliferation (**A** control vs shWBP2-1 vs shWBP2-2: 92 ± 5 vs 32 ± 5 vs 56 ± 6, *P* < 0.05), migration (**B** control vs shWBP2-1 vs shWBP2-2: 150 ± 11 vs 75 ± 8 vs 95 ± 2, *P* < 0.01), and invasiveness (**C** control vs shWBP2-1 vs shWBP2-2: 47 ± 5 vs 22 ± 2 vs 18 ± 3, *P* < 0.01) of lung cancer cells. In vivo: subcutaneous tumor transplantation and lung metastasis experiments via tail vein injection in nude mice reveal that volumes and weights of subcutaneously transplanted tumors in nude mice are significantly decreased (**D**, **E** control vs shWBP2-1: volume: 568.0 ± 125.3 vs 145.0 ± 9.3 [mm^3^], *P* < 0.05; weight: control vs shWBP2-1: 330.3 ± 49.2 vs 111.3 ± 7.5 [mg], *P* < 0.05), and the number of lung cancer metastasis foci is also significantly reduced (**F**, **G** control vs shWBP2-1: 6.1 ± 1.1 vs 2.4 ± 0.6, *P* < 0.01). *P* < 0.05 indicates statistical significance, *P < 0.05, **P < 0.01.

Next, we examined changes in the biological functions of lung cancer cells after WBP2 knockdown. We used lentivirus-coated shRNA-WBP2 to transfect the A549 cell line with high WBP2 expression. In contrast to the results of functional experiments, we observed diminished proliferative, migratory, and invasive abilities in A549 cells following WBP2 knockdown (Fig. 3A–C). In vivo, we observed that the volumes and weights of subcutaneous transplanted tumors, derived from A549 cells transfected with lentivirus-shRNA-WBP2 in nude mice, were significantly lower than those derived from control mice (Fig. 3D, E); the number of lung metastases induced by caudal vein metastasis was also significantly reduced (Fig. 3F, G). Therefore, combined with in vivo and in vitro experimental results, we can conclude that WBP2 functions as a tumor-promoting factor when exerting its potential biological functions in lung cancer cells.

### WBP2 is a negative regulator of the Hippo signaling pathway in lung cancer cells

**Fig. 4:**
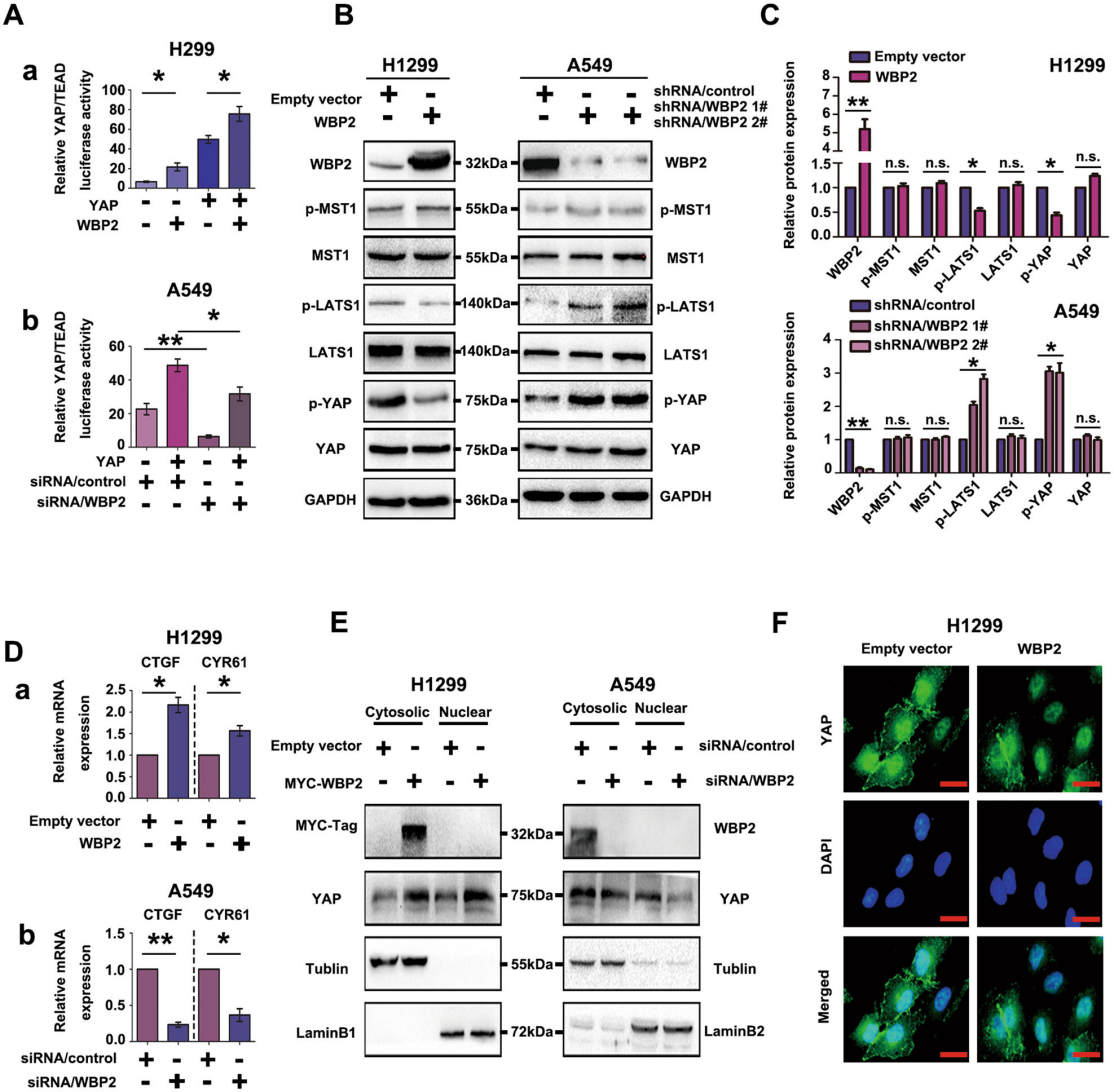
WBP2 is a negative regulator of the Hippo pathway in lung cancer cells. **A** (**a**, **b**) WBP2 upregulates the transcriptional activity of the TEAD promoter. The WBP2 plasmid and siRNA-WBP2 were transfected into H1299 and A549 cell lines, respectively. After 48 h, the cells were collected and lysed. Dual-luciferase reporter gene detection shows that the ectopic expression of WBP2 can significantly upregulate the transcriptional activity of TEAD (**A**–**a**), whereas it decreases after WBP2 knockdown (**A**–**b**). Transfection with YAP was used as the stimulus. TK was used as the internal reference. **B**, **C** WBP2 downregulates LATS1 phosphorylation and YAP phosphorylation through an MST-independent pathway. After overexpression and knockdown of WBP2 in H1299 and A549 cells, respectively, the phosphorylation and total amount of key proteins in the Hippo pathway were analyzed by western blotting. GAPDH was used as the loading control. Differences were analyzed using the Image J software. *P < 0.05, **P < 0.01. **D** (**a**, **b**), RT-qPCR analysis reveals that the mRNA levels of *CTGF* and *CYR61* are upregulated after WBP2 overexpression in H1299 (**D**–**a**), and the mRNA levels of these two target genes are downregulated (**D**–**b**) after WBP2 silencing in A549 cells. *P* < 0.05 indicates statistical significance, *P < 0.05, **P < 0.01. **E** Nuclear and cytosolic fractionation assays indicate that WBP2 overexpression promotes YAP nuclear translocation in H1299 cells, whereas WBP2 silencing in A549 exerts the opposite effect. Beta-tubulin and LaminB1 were used as the cytosolic and nuclear loading controls, respectively. **F** Immunofluorescence assay results reveal that the level of YAP nuclear translocation increases in response to WBP2 overexpression in H1299 cells. Magnification: ×400, scale bar: 50 μm.

At present, the underlying mechanism through which WBP2 affects the biological function of lung cancer cells remains unclear. We first examined the effect of WBP2 on the activity of the Hippo pathway in lung cancer cells. Initially, a dual-luciferase reporter assay was employed to demonstrate that WBP2 overexpression in H1299 significantly upregulated YAP-induced transcriptional activity in the TEAD reporter assay. Specifically, the activity of the Hippo pathway was inhibited (Fig. 4A–a). In contrast, the transcriptional activity of the TEAD reporter gene was downregulated by siRNA-WBP2 transfection in A549 cells, indicating that the Hippo pathway was activated (Fig. 4A–b). Phosphorylation of the MST-LATS complex in the Hippo pathway has a central role in classical Hippo activation. However, the effect of WBP2 on MST and LATS phosphorylation has not been previously elucidated. In the present study, western blot analyses revealed that phosphorylation levels of LATS1 and YAP were significantly downregulated in response to WBP2 overexpression; however, the phosphorylation levels and total amounts of MST did not demonstrate any significant changes (Fig. 4B, C), suggesting that WBP2 affected the phosphorylation of LATS1 and regulated the activity of the Hippo pathway in an MST-independent manner. RT-qPCR results revealed that transcription levels of target genes *CTGF* and *CYR61* were significantly upregulated after transfection with WBP2 (Fig. 4D–a), and the opposite was observed in response to WBP2 silencing (Fig. 4D–b). Furthermore, the GEPIA online correlation database (gepia.cancer-pku.cn) revealed a positive association between WBP2 and YAP target genes, including *CTGF*, *CYR61*, and *AREG* (Supplementary Fig. S3). Nuclear and cytosolic fractionation assays showed that ectopic WBP2 expression promoted YAP nuclear import in H1299 cells; conversely, WBP2 silencing exerted the opposite effect in A549 cells (Fig. 4E). Simultaneously, laser confocal detection assays revealed that the YAP level within the nucleus increased after transfection with WBP2 (Fig. 4F). These results indicated that WBP2 promotes YAP translocation into the nucleus by inhibiting LATS1 phosphorylation, ultimately inhibiting the activity of the Hippo pathway.

### Upstream protein WWC3 of the Hippo pathway was identified as a WBP2-binding protein

**Fig. 5:**
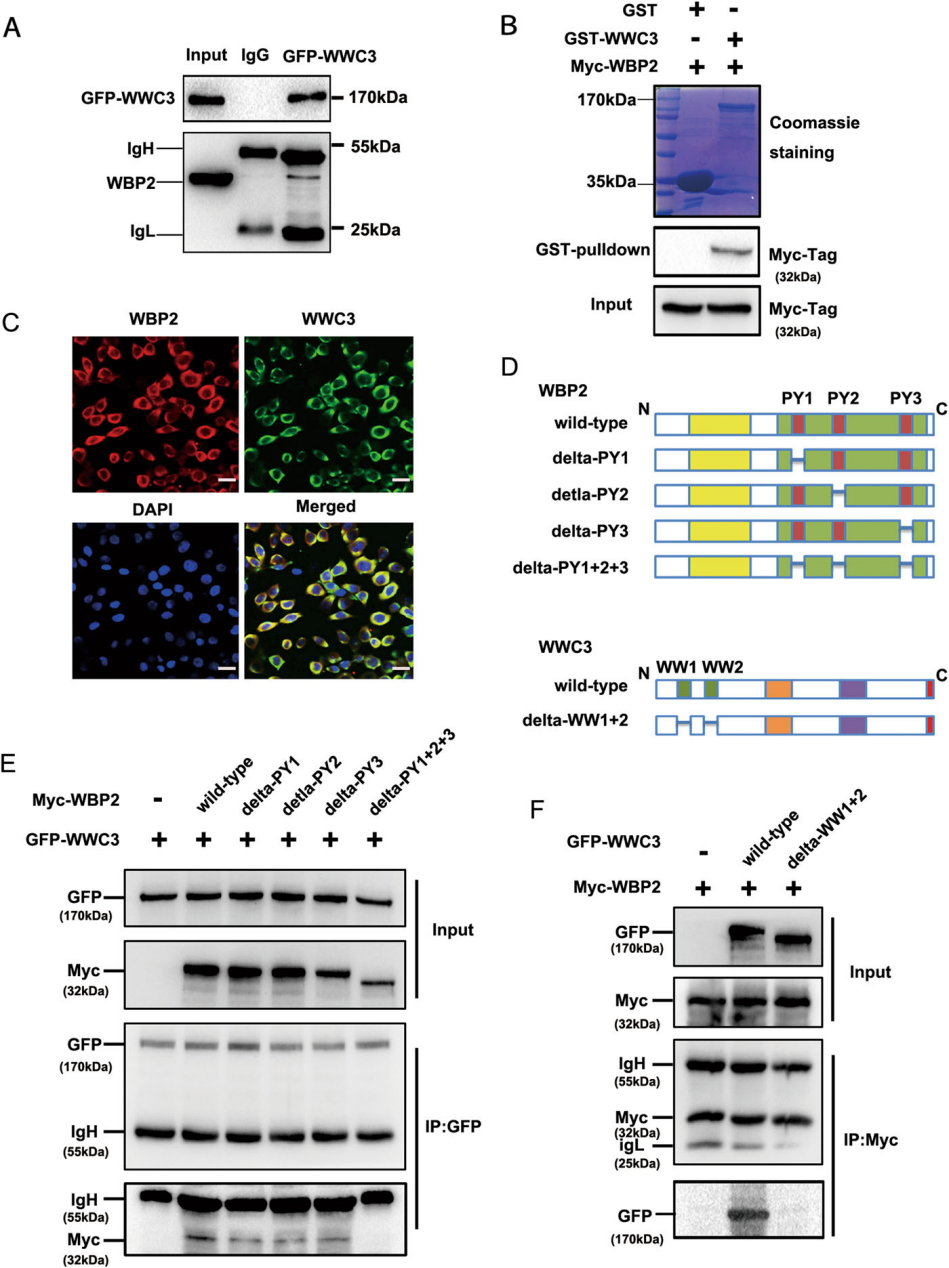
WBP2 interacts with the WW domain of WWC3 via PPxY motifs. **A** GFP-WWC3 was transfected into H1299 cells, and after 48 h, the cells were collected and lysed. A GFP monoclonal antibody was used for pulldown. The presence of WBP2 in the precipitate was detected by western blot analysis using a WBP2 antibody. **B** After incubation with purified GST or GST coupled-WWC3 protein for 6 h at 4° C, the binding status of two proteins was examined using Coomassie brilliant blue staining and western blot analysis. GST, glutathione-S-transferase. **C** Immunofluorescence assay results indicate that WBP2 and WWC3 colocalize within the cytoplasm of A549 cells. Magnification: ×400, scale bar: 50 μm. **D** Schematic diagram of WBP2 and WWC3 splicing mutants. **E** GFP-WWC3 and Myc-WBP2 wild-type or a series of mutants were co-transfected into H1299 cells. After 48 h, the cells were collected and lysed. GFP antibody was used for precipitation, and the presence of WBP2 was detected by immunoblotting using a Myc antibody. **F** Similarly, Myc-WBP2 and GFP-WWC3 wild-type or GFP-WWC3-△WW mutants were co-transfected into H1299 cells. GFP antibody was used for precipitation, and the presence of WBP2 was detected by immunoblotting using a Myc antibody.

Previous studies have suggested that WBP2 localizes within the nucleus of breast cancer cells and acts as a co-activator of YAP^21–24^. Interestingly, we observed that WBP2 was localized within the cytoplasm of lung cancer cells, and accordingly, we speculated that WBP2 may modulate the Hippo pathway in a YAP-indirect-dependent manner. This knowledge, combined with our previous results, indicated that WBP2 can downregulate LATS1 phosphorylation levels, and therefore, we examined the upstream protein molecules that can cause changes in LATS1 phosphorylation levels. The WWCs protein family contains the classical upstream molecules of the Hippo pathway that can interact with LATS1 and promote LATS1 phosphorylation^17,18^. Immunoprecipitation and GST-pulldown assays verified that WBP2 dramatically interacted with the upstream protein WWC3, with both interacting directly (Fig. 5A, B). Confocal laser scanning revealed that WBP2 and WWC3 colocalized in the cytoplasm of A549 cells that possess high expression levels of both proteins (Fig. 5C). To further explore the structural basis underlying the binding of WBP2 and WWC3^18^, we constructed a series of WBP2 (deletion of PPxY motifs) and WWC3 (deletion of double WW domains) mutants (Fig. 5D), and then transfected them into H1299 cells, as these cells exhibited high transfection efficiency. In addition, immunoprecipitation studies indicated that WBP2 was bound to the WW domain of WWC3 through its PPxY motifs (Fig. 5E, F).

### WBP2 inhibits the activity of the Hippo pathway by inhibiting LATS1 phosphorylation via interaction with WWC3

**Fig. 6:**
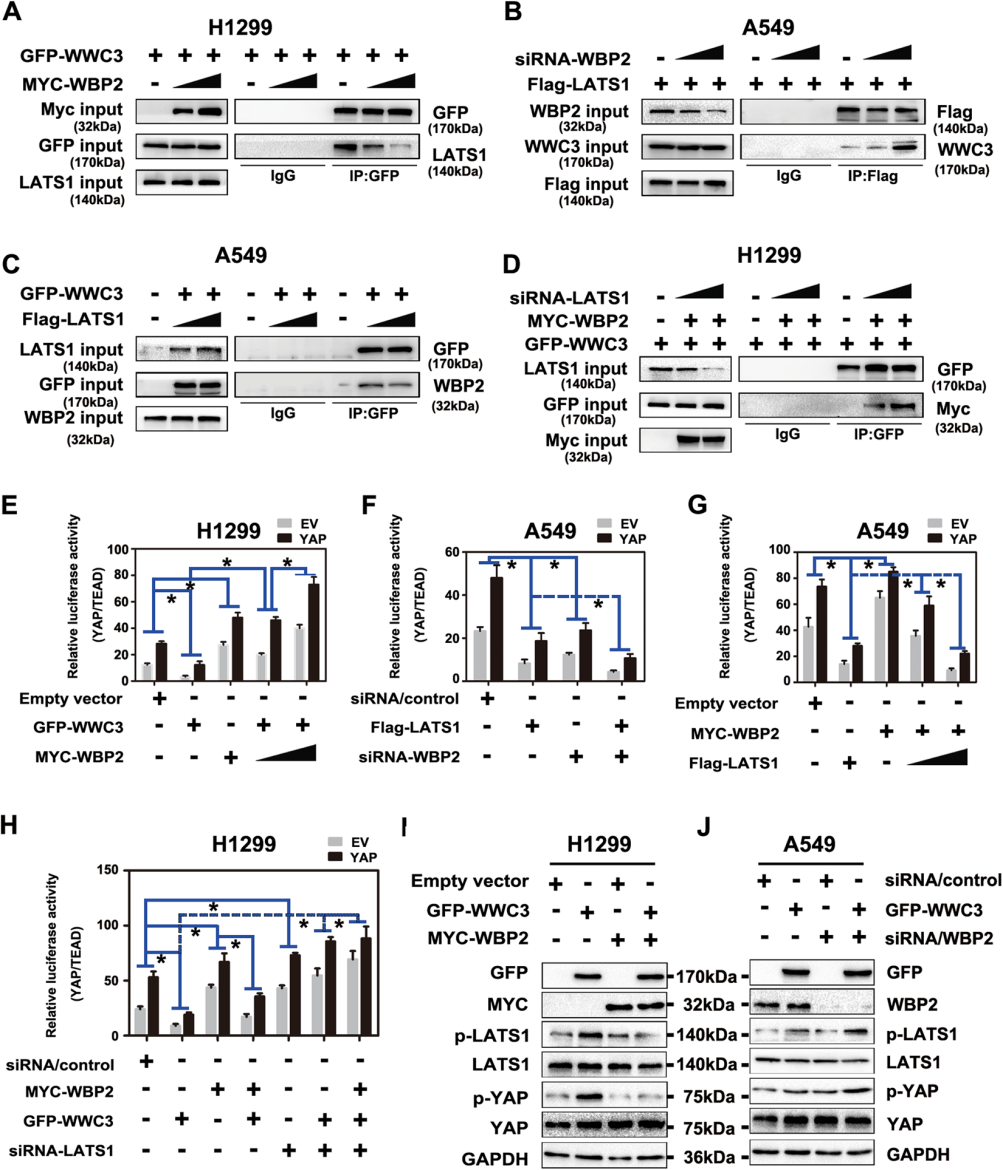
WBP2 and LATS1 competitively bind to WWC3 to inhibit Hippo pathway activity. **A** WBP2 overexpression attenuates the binding between WWC3 and LATS1. In the H1299 cell line, the WBP2 plasmid (1 μg and 2.5 μg) was transfected in a dose gradient manner. After 48 h, the cells were collected and lysed. A GFP monoclonal antibody was used for immunoprecipitation. The combination change in WWC3 and LATS1 protein in the sediment was detected by western blotting. **B** The binding of WWC3 to LATS1 increases after WBP2 knockdown. siRNA-WBP2 (5 pmol and 10 pmol) was transfected into A549 cells with high WBP2 expression. After cell collection, binding was detected by anti-FLAG immunoprecipitation followed by anti-WWC3 immunoblotting. **C**, **D** Following transfection of LATS1 into A549 cells with low LATS1 expression, the binding of WBP2 to WWC3 gradually decreases with an increase in LATS1 expression (**C**). In contrast, the binding of WBP2 to WWC3 gradually increases with the downregulation of LATS1 after siRNA-LATS1 was transfected into H1299 cells with high LATS1 expression (**D**). **E**, **F** Forty-eight hours post-transfection with WBP2 plasmid or siRNA-WBP2 into H1299 cells and A549 cells, the cells were collected and lysed. The results of the dual-luciferase reporter assay reveal that WBP2 overexpression reverses the decrease in TEAD transcriptional activity induced by WWC3 (**E**). Conversely, WBP2 knockdown further enhances the decrease in TEAD transcriptional activity induced by WWC3 (**F**). **G**, **H** Similarly, dual-luciferase reporter gene detection assays reveal that LATS1 overexpression reverses the WBP2-induced increase in TEAD transcriptional activity (**G**). Moreover, silencing of LATS1 further enhances the WBP2-induced increase in TEAD transcriptional activity (**H**). **I**, **J** Western blot analysis indicates that overexpression of WBP2 reverses the increase in LATS1 and YAP phosphorylation levels induced by WWC3 (**I**). Conversely, knockdown of WBP2 further increases the phosphorylation levels of LATS1 and YAP (**J**). *P* < 0.05 indicates statistical significance, *P < 0.05.

WWC3 can activate the Hippo pathway by interacting with LATS1 kinase via the WW domain, promoting LATS1 phosphorylation. Moreover, the PPxY motifs of WBP2 can combine with the WW domain of WWC3; accordingly, we speculated whether WBP2 competitively binds to the WW domain of WWC3 via LATS1 to weaken the activity of the WWC3-LATS1 complex. To verify this hypothesis, we first increased the expression of WBP2 in H1299 cells exhibiting low WWC3 and WBP2 expressions and high LATS1 expression^25,26^. In these cells, immunoprecipitation experiments revealed that the binding of WWC3 and LATS1 gradually decreased in response to WBP2 overexpression in a dose-dependent manner (Fig. 6A). In contrast, the binding ability of LATS1 to WWC3 dramatically increased after WBP2 knockdown in A549 cells (low expression of LATS1 and high expression of WWC3 and WBP2) (Fig. 6B). Conversely, we overexpressed LATS1 in A549 cells and observed that the binding of WBP2 and WWC3 gradually decreased (Fig. 6C). However, after LATS1 was knocked down in H1299 cells, the binding of WBP2-WWC3 gradually increased in a dose-dependent manner (Fig. 6D). These results demonstrated that WBP2 competitively binds to WWC3 with LATS1. Next, we explored the impact of this competitive interaction between these three factors on the Hippo pathway. First, using a dual-luciferase reporter assay and qRT-PCR, we observed that the ectopic expression of WBP2 in H1299 cells significantly rescued the decrease in YAP-TEAD transcriptional activity and Hippo pathway target gene expressions (*CTGF* and *CYR61*) induced by WWC3 (Fig. 6E, Supplementary Fig. S4-A). However, following WBP2 knockdown in A549 cells, the inhibition of Hippo induced by LATS1 was further promoted (Fig. 6F, Supplementary Fig. S4-B). In contrast, overexpression of LATS1 dramatically reduced the increase in YAP-TEAD activity and Hippo pathway target genes induced by WBP2 (Fig. 6G, Supplementary Fig. S4-C); this effect was reversed after LATS1 knockdown (Fig. 6H, Supplementary Fig. S4-D). Accordingly, western blot analyses also revealed that WBP2 overexpression significantly reversed the upregulation of LATS1 and YAP phosphorylation induced by WWC3 in H1299 cells; this effect was abrogated following WBP2 knockdown (Fig. 6I, J). Based on the above results, we believe that WBP2 competes with LATS1 to bind to WWC3, and this competitive binding results in a decrease in WWC3-LATS binding, eventually resulting in the downregulation of LATS1 phosphorylation to inhibit the activity of the Hippo pathway.

## Discussion

A growing number of studies have reported the carcinogenic effect of WBP2 in human solid tumors, particularly breast cancer^11–15,27–29^. In the present study, we observed that WBP2 was highly expressed in NSCLC and associated with poor prognosis in patients. However, the underlying cause of high WBP2 expression in lung cancer remains unclear. Based on existing literature, WBP2 expression could be affected by several underlying factors, including non-coding micro-RNA^30^, transcriptional factors^31^, and ITCH^32^, as well as some classical signaling transduction pathway activities^33^. These experimental data have helped clarify the reasons for high WBP2 expression in different tumors. Regarding the high WBP2 expression in lung cancer, it remains unclear whether this is related to the amplification of the gene promoter, transcription level, or post-translation regulation. Accordingly, these factors need to be explored in follow-up investigations.

In NSCLC, inactivation of the Hippo pathway is a common phenomenon that is closely related to the occurrence, development, and drug resistance of lung cancer^34^. We verified that WBP2 competitively binds to the WW domain of WWC3 via LATS1 to inhibit the formation of the WWC3-LATS1 complex and reduce the phosphorylation level of LATS1, promoting YAP translocation into the nucleus. This inhibits the Hippo pathway and ultimately promotes the malignant phenotype of lung cancer (Supplementary Fig. S5). Herein, WBP2 regulated Hippo activity in a unique manner that was not directly dependent on YAP at the upstream level. Notably, deletion of WW domains of WWC3 and PPxY motifs of WBP2 might result in conformational changes of both proteins. Adopting CRISPR/Cas9 editing of WW domains or PPxY in endogenous genes/proteins to create point mutations is much better to render WW domains and PPxY motifs inactive in terms of binding.

The WWC protein family is highly conserved in evolution. The other two members, WWC1/KIBRA and WWC2, are highly similar to WWC3 in molecular structure and contain double WW domains. Recent reports have indicated that WWC1 and WWC2 can function as tumor suppressor molecules in lung cancer^35,36^. Similarly, LATS2, a homolog of LATS1, has been largely studied in NSCLC. For example, LATS2 is reportedly expressed in NSCLC and is closely related to poor patient prognosis and chemotherapy resistance^37,38^. Herein, WWC1/KIBRA, WWC2, and LATS2 (also containing PPxY motifs) might coordinate with WBP2 to have an essential role in NSCLC progression, which will be an interesting direction for our future research. Moreover, WBP2 is an interacting protein of YAP, which can function as a co-activator to enhance its effect. TAZ, a homolog of YAP, is closely associated with the function of WBP2. In non-tumor cells, MCF-10A and NIH3T3, the binding of WBP2 and TAZ is vital for cell proliferation^39^. Combined with the implicated role of TAZ in lung cancer, the crosstalk between WBP2 and TAZ in lung cancer also warrants further investigation.

## Supplementary information

Supplementary figure legends

Supplementary material and methods

Supplementary Figure S1

Supplementary Figure S2

Supplementary Figure S3

Supplementary Figure S4

Supplementary Figure S5
