# Supplementary material for: WBP2 negatively regulates the Hippo pathway by competitively binding to WWC3 with LATS1 to promote non-small cell lung cancer progression
Source: Cell Death Dis. 2021 Apr 9;12(4):384. doi: 10.1038/s41419-021-03600-3 (PMC8035140; doi:10.1038/s41419-021-03600-3)
Supplement: Supplementary file 1 — Supplementary figure legends [file 41419_2021_3600_MOESM1_ESM.docx]

**Supplementary Figure S1.** Laser confocal indicated WBP2 was located in the cytoplasm of lung cancer cell lines. Magnification: 400×; Scale Bar: 50 μm.

**Supplementary Figure S2.** Survival analysis showed that the survival time of patients with WBP2-high staining lung cancer was significantly shorter than that of patients with negative or low expression.

**Supplementary Figure S3.** Positive correlation between WBP2 and *CTGF*, *Cyr61,* and *AREG* in lung cancer, analyzed at the GEPIA website.

**Supplementary Figure S4.** **A-B:** After 24 hours post transfection with WBP2 plasmid or siRNA-WBP2 into H1299 cells and A549 cells, qRT-PCR assay revealed that WBP2 overexpression reversed the decrease in *CTGF* and *CYR61* mRNA expression caused by WWC3 (**A**). Conversely, WBP2 knockdown further enhanced the decrease in *CTGF* and *CYR61* mRNA expression caused by WWC3 (**B**).

**C-D:** Similarly, LATS1 overexpression reversed the WBP2-induced increase in *CTGF* and *CYR61* mRNA expression (**C**). Moreover, silencing of LATS1 further enhanced the WBP2-induced increase in *CTGF* and *CYR61* mRNA expression (**D**).

*P <* 0.05 indicates statistical significance, * *P <* 0.05, ** *P <* 0.01.

**Supplementary Figure S5.** Schematic diagram of the molecular mechanism of WBP2-mediated regulation of the Hippo signaling pathway
