# Supplementary material for: WBP2 negatively regulates the Hippo pathway by competitively binding to WWC3 with LATS1 to promote non-small cell lung cancer progression
Source: Cell Death Dis. 2021 Apr 9;12(4):384. doi: 10.1038/s41419-021-03600-3 (PMC8035140; doi:10.1038/s41419-021-03600-3)
Supplement: Supplementary file 2 — Supplementary material and methods [file 41419_2021_3600_MOESM2_ESM.docx]

*Antibodies for immunoboltting*

The primary antibodies included WBP2 (#11831, IB/1:1000), Myc-Tag (#2276, IB/1:1000, IP/1:50), FLAG-Tag (#14793, IB/1:1000, IP/1:50), MST (#3682, IB/1:1000), p-MST (#49332, IB/1:1000), p-LATS1 (#8654, IB/1:1000), LATS1 (#3477, IB/1:1000), p-YAP (#13008, IB/1:1000), YAP (#14074, IB/1:1000), and actin (#3700, IB/11000) and were purchased from Cell Signaling Technology. Inc. (Danvers, MA, USA). WWC3 (HPA039814, IB/1:500) was purchased from Sigma-Aldrich (St. Louis, MO, USA), GAPDH (sc-293335, IB/1:1000) was purchased from Santa Cruz Biotechnology Inc. (CA, USA), and GFP-Tag (#JL-8, IB/1:3000) was purchased from Clontech (TaKaRa Bio, Beijing, China).

*Plasmids and small interfering RNA*

Myc/DDK-tagged-pCMV6 empty vector and Myc/DDK-tagged-pCMV6-WBP2 plasmids were purchased from Origene (product No. PS-100001 and RC-203955, respectively, Rockville, MD, USA). To construct the human Myc/DDK-tagged-pCMV6-WBP2-deltaPY1 (amino: Del167-170) expression plasmid, the coding sequence was amplified with primers: Forward：5’-ATGGAATGTACCCCTGCCCCTATCCACCGCCCCCAC-3’, Reverse: 5’-GTGGGGGCGGTGGATAGGGGCAGGGGTACATTCCAT-3’; For Myc/DDK-tagged-pCMV6-WBP2-deltaPY2 (amino: Del197-200) expression plasmid, the primers were: Forward: 5’-GGATACGTGCAGCCCCCACCTGGGCCCATGGAACCTC-3’, Reverse: 5’- GAGGTTCCATGGGCCCAGGTGGGGGCTGCACGTATCC-3’; For Myc/DDK-tagged-pCMV6-WBP2-deltaPY3 (amino: Del: 249-252) expression plasmid, the primers were: Forward： 5’-GCCCACGAGCCAGCCGCCGCCACCGGAAGATAAGAAG-3’, Reverse: 5’-CTTCTTATCTTCCGGTGGCGGCGGCTGGCTCGTGGGC-3’. The Myc/DDK-tagged-pCMV6-WBP2-deltaPY1+2+3, pcDNA3.1 empty vector and pcDNA3.1-FLAG-LATS1 were constructed by BaiHao Biotechnology Company (Shenyang, China). pEGFP-C2 empty vector, p-EGFP-C2-WWC3 and p-EGFP-C2-WWC3-deltaWW and were provided by Professor Joachim Kremerskothen of Muenster University, Germany. All constructs were verified by sequencing. ShRNA-control, shRNA-WBP2-1#, shRNA-WBP2-2#, and shRNA-WBP2 were coated with lentivirus by the Genechem Company (Shanghai, China). Control siRNA (sc-37007), siRNA-WBP2 (sc-93955), and siRNA-LATS1 (sc-35546) were purchased from Santa Cruz Biotechnology. p2xFlag-YAP1 (#17791) and pGL3b_8xGTIIC-luciferase (#34615) were purchased from Addgene (Cambridge, MA, USA). The pRL-TK vector (#E2241) was purchased from Promega (Madison, WI, USA).

*Quantitative PCR*

The primers for real-time reverse transcriptase polymerase chain reaction were:WBP2: 5′-CAAGAGCGCATGTTGAAGGAA-3′, 5′-CGCTGCTGCTTAATCTGGTAGA-3′; LATS1: 5′-AATTTGGGACGCATCATAAAGCC-3′, 5′-TCGTCGAGGATCTTGGTAACTC-3′; WWC3: 5′-CAAGAGCGCATGTTGAAGGAA-3′, 5′-CGCTGCTGCTTAATCTGGTAGA-3′; CTGF: 5′-AACTGCAACCTCTCGCACTG-3′, 5′-GCTCGGGCTCCTTGTAATTCT-3′; CYR61: 5′-CTCGCCTTAGTCGTCACCC-3′, 5′-CGCCGAAGTTGCATTCCAG-3′; GAPDH: 5′-GGAGCGAGATCCCTCCAAAAT-3′,5′-GGCTGTTGTCATACTTCTCATGG-3′.
