# Supplementary figures and images for: WBP2 negatively regulates the Hippo pathway by competitively binding to WWC3 with LATS1 to promote non-small cell lung cancer progression
Source: Cell Death Dis. 2021 Apr 9;12(4):384. doi: 10.1038/s41419-021-03600-3 (PMC8035140; doi:10.1038/s41419-021-03600-3)

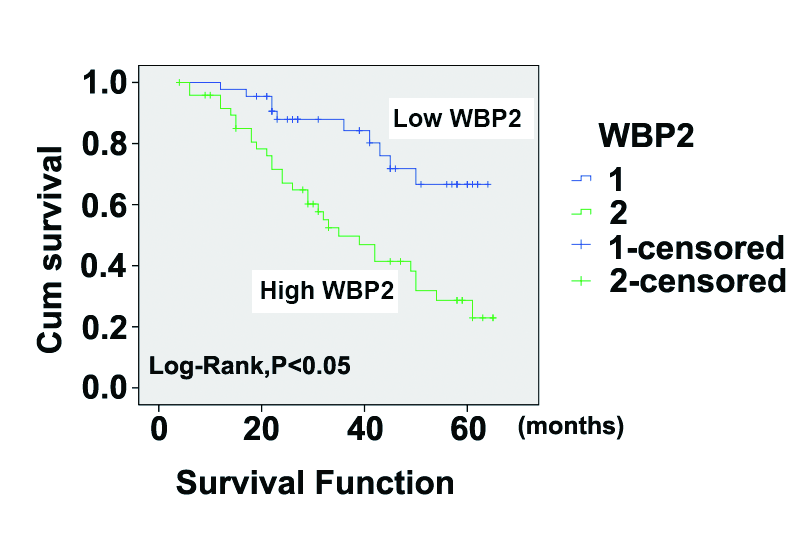

Supplement: Supplementary file 3 — Supplementary Figure S1 [file 41419_2021_3600_MOESM3_ESM.tif]

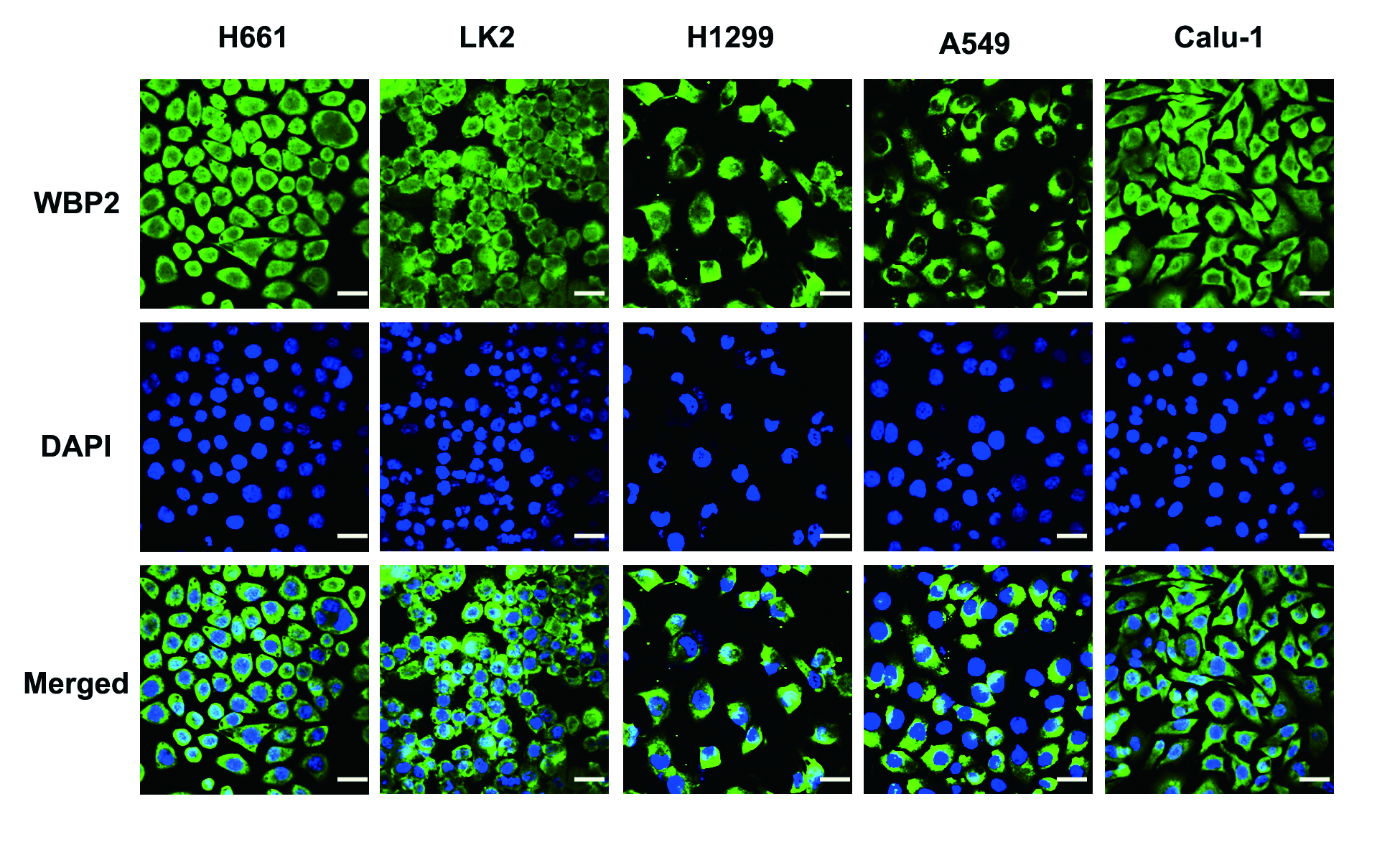

Supplement: Supplementary file 4 — Supplementary Figure S2 [file 41419_2021_3600_MOESM4_ESM.tif]

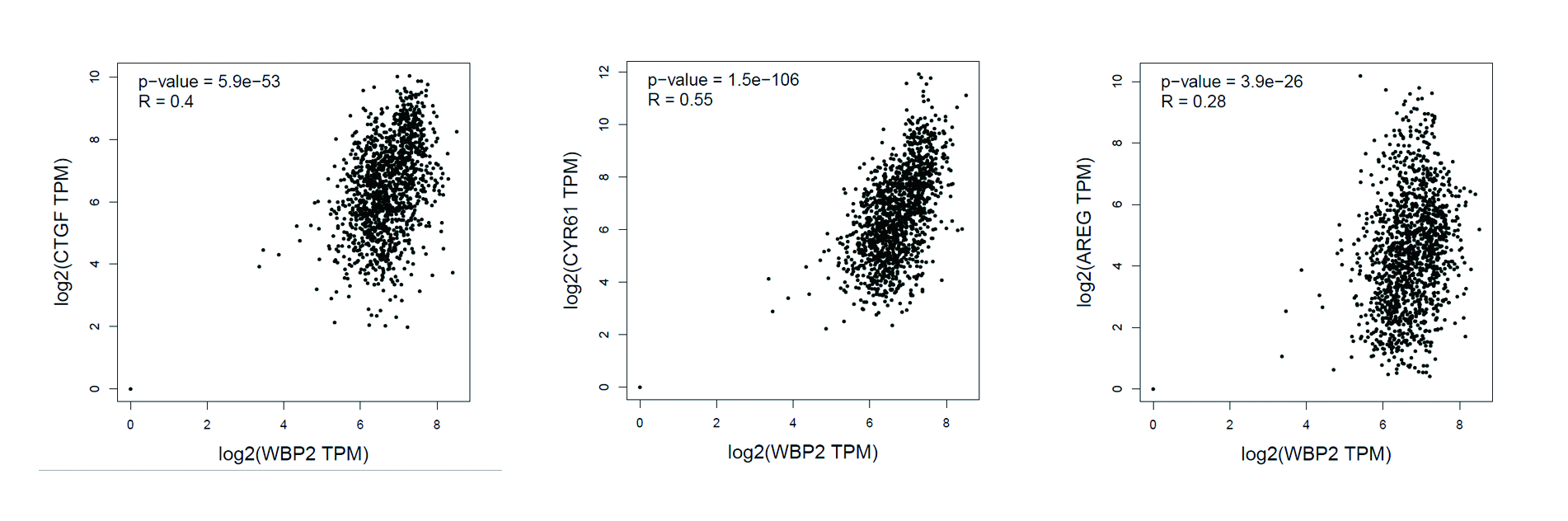

Supplement: Supplementary file 5 — Supplementary Figure S3 [file 41419_2021_3600_MOESM5_ESM.tif]

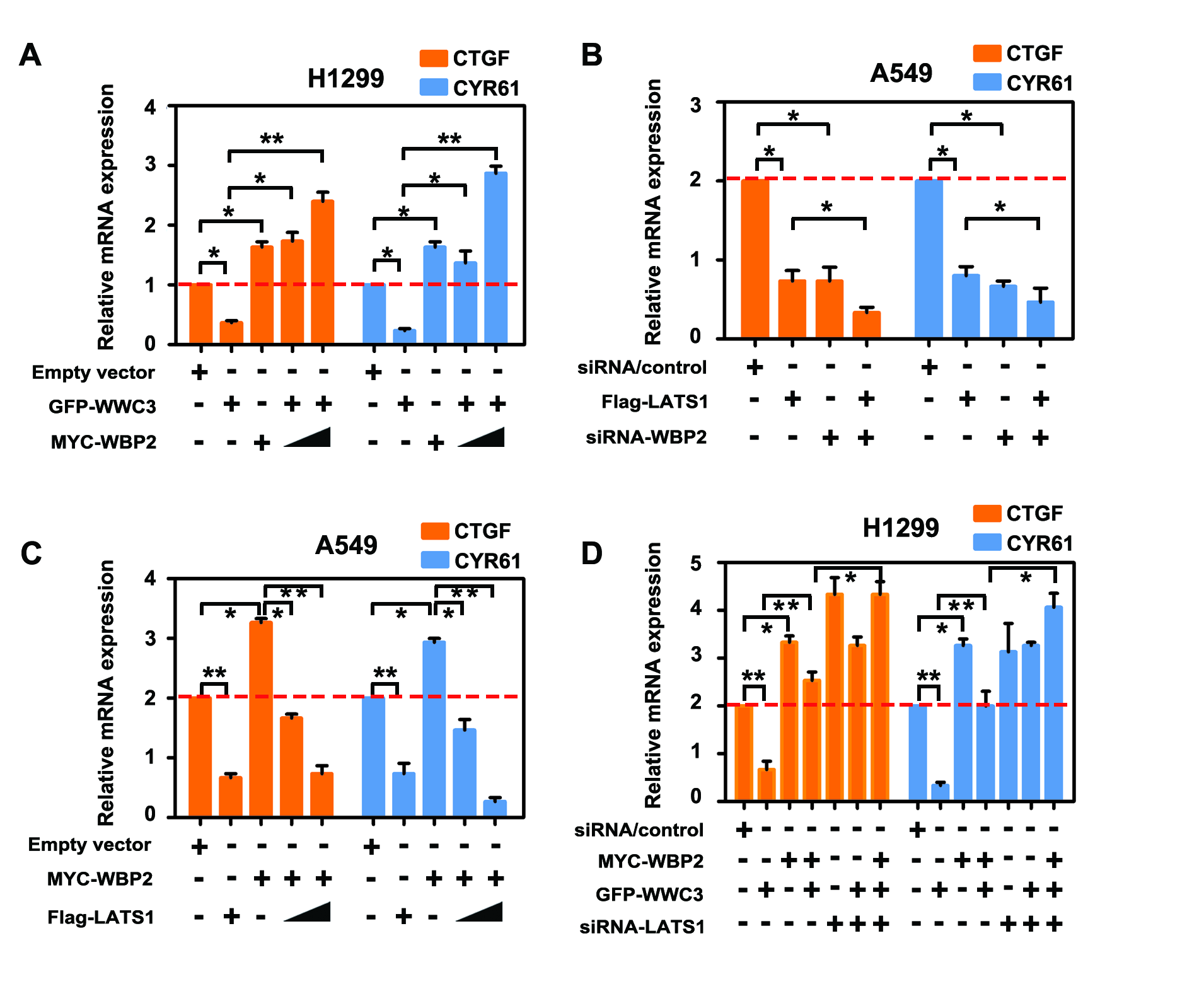

Supplement: Supplementary file 6 — Supplementary Figure S4 [file 41419_2021_3600_MOESM6_ESM.tif]

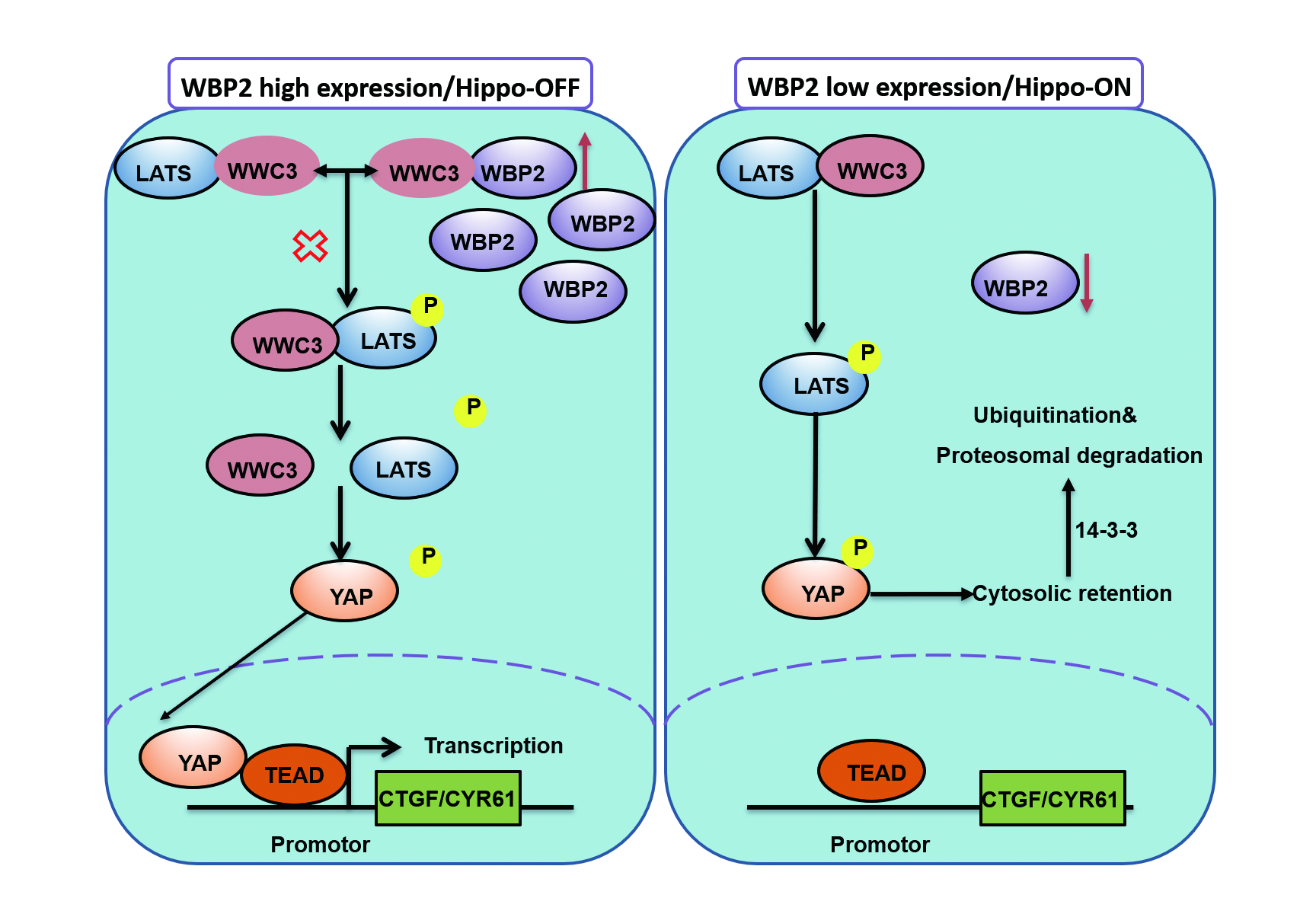

Supplement: Supplementary file 7 — Supplementary Figure S5 [file 41419_2021_3600_MOESM7_ESM.tif]
